# Epidemiological, Clinical, Ultrasonographic and Cytological Characteristics of Thyroid Nodules in an Afro-Caribbean Population: A Series of 420 Patients

**DOI:** 10.3390/cancers14102365

**Published:** 2022-05-11

**Authors:** Elodie Rano, Lucien Lin, Vincent Molinie, Caroline Sulpicy, Marie-Josée Dorival, Kinan Drak Alsibai, Mathieu Nacher, Moustafa Drame, Nadia Sabbah

**Affiliations:** 1 Department of Endocrinology and Metabolic Diseases, University Hospital Centre Louis Domergues, F-97220 La Trinité, France; elodie.rano@gmail.com (E.R.); linluc@wanadoo.fr (L.L.); 2 Department of Pathology, University Hospital Centre Pierre Zobda Quitman, F-97261 Fort-de-France, France; vmolinie@gmail.com; 3 Pathology Centre, F-97233 Schoelcher, France; csulpicy@wanadoo.fr (C.S.); mjdorival@wanadoo.fr (M.-J.D.); 4 Centre of Biological Resources (CRB Amazonie), Cayenne Hospital Centre, F-97306 Cayenne, French Guiana; kdrak.alsibai@doctor.com; 5 Clinical Investigation Center Antilles-French Guiana (CIC, INSERM 1424) Cayenne Hospital Centre Andrée Rosemon, F-97306 Cayenne, French Guiana; mathieu.nacher@ch-cayenne.fr; 6 Department of Medical Information, University Hospital Centre Pierre Zobda Quitman, F-97261 Fort-de-France, France; moustafa.drame@chu-martinique.fr; 7 Department of Endocrinology and Metabolic Diseases, Cayenne Hospital Centre Andrée Rosemon, F-97306 Cayenne, French Guiana

**Keywords:** thyroid cancer, thyroid nodule, Bethesda system, cytopathology, ultrasound, Afro-Caribbean population, management

## Abstract

**Simple Summary:**

This study describes the epidemiological, clinical and ultrasound characteristics of malignancy in thyroid nodules and evaluates the value of cytology in the diagnosis of malignancy in an Afro-Caribbean population. Our results revealed that none of the standard ultrasound criteria of malignancy were significantly predictive of cancer, but hypoechogenicity and central vascularity were frequently found in malignant nodules. These results could increase awareness and guide practitioners in their diagnostic approach and management of thyroid nodules in Afro-Caribbean populations. Bethesda system-based cytology revealed low sensitivity in analyzing the risk of malignancy in this population. The high prevalence of papillary microcarcinomas may explain the inconclusive ultrasound and cytological results.

**Abstract:**

The incidence of thyroid cancer is increasing worldwide. The aim of this study is to describe the epidemiological, clinical and ultrasound characteristics of malignancy in thyroid nodules and to evaluate the predictive value of the Bethesda system for thyroid cytology in the diagnosis of malignancy in an Afro-Caribbean population. We conducted a retrospective study in Martinique involving 420 patients with a diagnosis of thyroid nodules between 2011 and 2014. Of the 192/420 (45.7%) patients operated on for thyroid nodules, 9% had thyroid cancer. All patients with thyroid cancer were obese women with a mean age of 50 years. The final histological examination revealed papillary microcarcinomas in 61% of cases and papillary carcinomas in 39% of cases. Thyroid cytology alone had a low sensitivity (22.2%) and positive predictive value (15.4%) for the diagnosis of malignancy, with a good specificity (91.1%) and negative predictive value (94.2%). None of the standard ultrasound criteria of malignancy were significantly predictive of cancer, but hypoechogenicity and central vascularity were frequently found in malignant nodules. These epidemiological, clinical and ultrasound results could increase awareness and guide practitioners in their diagnostic approach and management of thyroid nodules in an Afro-Caribbean population. Bethesda system-based cytology revealed lower sensitivity in analyzing the risk of malignancy in this population. The high prevalence of papillary microcarcinomas may explain the inconclusive ultrasound and cytological results.

## 1. Introduction

In general practice, the discovery of a thyroid nodule is a frequent motive for consultation. The prevalence of palpable thyroid nodules is about 5%, whereas in the general population, occult nodules could reach 68% [1]. Nodules are sometimes discovered by cervical palpation and may be accompanied by clinical signs such as dyspnea or swallowing disorders [1,2]. Cervical ultrasound is the reference examination of thyroid lesions. It allows the characterization of the nodules and classifying them according to the European Thyroid Imaging Reporting and Data System (EU-TIRADS classification) [3]. Thyroid fine-needle aspiration (FNA) cytology is used to determine the risk of malignancy (Bethesda classification 2017), by taking into consideration the clinical and ultrasound malignancy criteria [4,5]. Other radiological examinations such as cervical computed tomography (CT), positron emission tomography (PET) and magnetic resonance imaging (MRI) are rarely performed for the primary evaluation of thyroid nodules. These methods can be used in the extension assessment of a suspicious or malignant nodule or in the context of a second line of treatment [6]. Fortunately, 95% of thyroid nodules are benign [7]. However, there is an increasing incidence of thyroid cancer worldwide [8]. Although thyroid cancer generally has a good prognosis, the increasing incidence raises the question of the factors that encourage the occurrence of these cancers. In the literature, the recognized risk factors for thyroid malignancy are: a history of cervical irradiation or accidental exposure to ionizing radiation in childhood or adolescence, a family history of thyroid cancer or hereditary syndromes that include genetic predisposition, firm, fixed or rapidly growing nodules, hypoechogenicity, irregular contours, incomplete halo, hypervascularization and microcalcifications on ultrasound examination, and the presence of enlarged cervical lymph nodes [9]. Other less specific clinical signs which may raise suspicion of thyroid malignancy are: male, child or elderly subjects, presence of compressive signs and nodules measuring more than 3 cm [10].

A previous study of thyroid cancer in Martinique revealed a clear predominance of women without a previous history [2]: 9% of operated nodules were histologically malignant, including 50% of papillary carcinomas and 30% of papillary microcarcinomas. In 11% of cases, the cytological results did not correspond to the final histological analyses, which raised the question of the predictive value of the Bethesda system in the FNA cytological diagnosis of malignancy in this population. Other teams have also looked at the value of the standardized Bethesda classification for the diagnosis of thyroid malignancy and revealed a difference between African-American and Caucasian populations [11].

The aim of this study was to describe the epidemiological, clinical and ultrasonographic characteristics of malignancy of thyroid nodules and to evaluate the relevance of the Bethesda system in an Afro-Caribbean population in order to optimize their management in general practice.

## 2. Materials and Methods

We conducted a retrospective study from 2011 to 2014 including 525 adult patients from the Department of Endocrinology (University Hospital Centre, La Trinité, Martinique, France). The Department of Endocrinology is the referent center of thyroid cytopunction in Martinique. All included patients had at least one thyroid nodule with FNA cytological analysis. Patients referred to our department for thyroid ultrasound received an ultrasound-guided FNA if the nodule measured more than 8 mm, with at least one clinical malignancy criterion (firm on clinical examination, presence of cervical nodes on palpation) and/or signs of ultrasound malignancy: hypoechogenicity, irregular contours or microcalcifications. We also used the presence of central vascularity as a sign of malignancy (a criterion used in ultrasound reports of our department). Thyroid nodules classified as benign were punctured when they measured more than 1 cm.

Based on the following criteria, 105 patients were excluded: patients under 18 years of age, those without FNA analysis or an incomplete file with many missing data points.

Ultrasound and ultrasound-guided FNA were performed with a Philips-Advanced Technology Laboratories device and a 5 to 12 MHz linear transducer by two endocrinologists with more than ten years of experience in thyroid ultrasound.

Ultrasound characteristics were size, location, content (liquid, solid, mixed), structure (homogeneous, heterogeneous), echogenicity, contours (regular, irregular), presence or absence of a halo or microcalcifications. Vascularity was classified as type I if absent, type II for a predominantly peripheral flow, type III for central flow and type IV for an anarchic flow (central and peripheral important).

The TIRADS classification was not yet used in our department during the study period because it was implemented in 2016. As the “higher than wide” shape and rigidity were not described on ultrasound, we could not establish correspondence with the TIRADS or EU-TIRADS classification currently used in clinical practice.

Patients signed an informed consent before the FNA was performed. Local anesthesia was applied before fine-needle aspiration with 1% xylocaine gel. Samples were taken with a 25 G needle and a 10 mL syringe, if necessary, then spread on slides, fixed with alcohol and subjected to Papanicolaou staining. When the nodule had mixed contents (cystic and solid), both components were taken. The slides were then sent to the Pathology Department of the University Hospital Centre of Martinique.

Cytological findings were classified according to the Bethesda system 2010 into six categories [12]: I: Not satisfactory for diagnosis, II: Benign, III: Follicular lesion of undetermined significance or atypia of undetermined significance, IV: Follicular neoplasia or suspected follicular neoplasia (including Hürthle cells neoplasia), V: Suspicious of malignancy and VI: Malignant.

Patients with clinically and sonographically suspicious nodules, and/or measuring more than 3 cm, and/or with a Bethesda V and VI or rechecked Bethesda IV, were presented to a multidisciplinary thyroid consultation, for a surgical management decision (unless contraindication of surgery). The surgical specimen was sent to the Department of Pathology (University Hospital Centre, Fort-de-France, Martinique, France) or to the Pathology Centre (Schoelcher, Martinique, France).

### 2.1. Data Collection

The data were obtained from patient records archived in The Department of Endocrinology—Martinique Hospital Centre, and from the laboratory software. The following clinical parameters were collected: social and demographic data, gender, age, geographical area, referring physician, personal or family history of thyroid pathology (nodule, thyroidectomy or lobectomy, dysthyroidism), radiation, weight, height and body mass index (BMI) and clinical examination including signs of clinical dysthyroidism (hypothyroidism/hyperthyroidism). Collected biological data include: TSH (Thyroid Stimulating Hormone) and FT4 (Free Thyroxine) analyses, antithyroid autoantibodies, anti-thyroperoxidase (AcTPO), anti-thyroglobulin (AcTG), if TSH > 4.5 miU/L or if appearance of thyroiditis on ultrasound, and anti-TSH receptor antibodies (TRAK) (if TSH < 0.4 miU/L). Ultrasound, cytological and histological results were also recorded.

### 2.2. Ethical Statement

This study has obtained authorization from the Department of Medical Information of Centre Hospitalier Universitaire de Martinique and the National regulatory authority, Commission Nationale de l’Informatique et des Libertés (CNIL) (declaration No. 2213479).

### 2.3. Statistical Analysis

SAS software, version 9.4 (SAS Institute, Inc., Cary, NC, USA), was used for statistical analysis. The analysis of quantitative variables required calculations of mean and standard deviation in order to perform tests of equality of variances of two sub-populations, and tests of equality of means. For categorical variables, chi-square and Fisher tests were used. A diagnostic test was used to evaluate the effectiveness of the FNA cytology via calculations of sensitivity, specificity, positive and negative predictive values. If a focus of microcarcinoma was found outside of the nodule of interest, it was considered clinically insignificant and was excluded for determining the predictive value of the FNA cytology.

We considered patients with malignant histology as “diseased” and those with benign histology as “not diseased”. Cytology results classified as Bethesda V and VI were considered as high risk for malignancy and those classified as Bethesda I and II as low risk of malignancy, while Bethesda III and IV were considered as medium risk of malignancy. A value of *p* < 0.05 was taken as statistically significant.

## 3. Results

### 3.1. Description of the General Population

**Figure 1:**
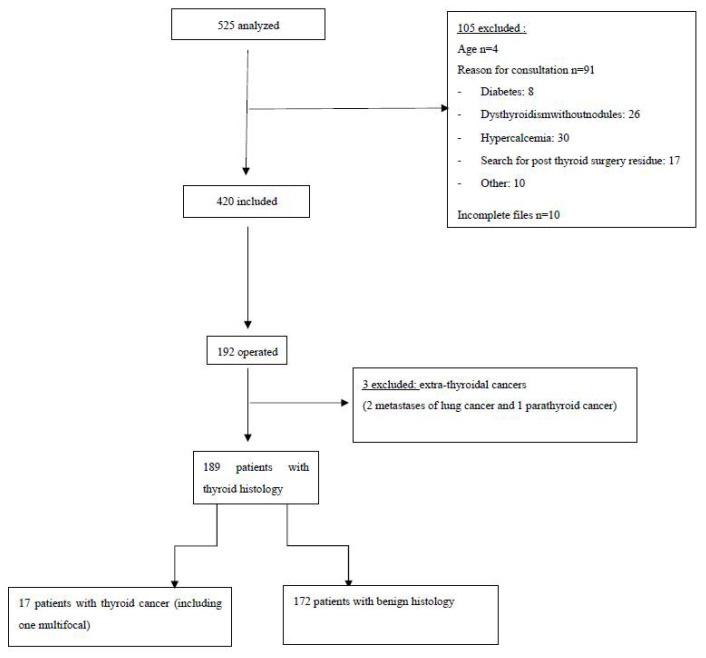
Flow diagram.

Between 2011 and 2014, 525 patients with thyroid nodules were managed in our Department of Endocrinology, and 105 patients were excluded from this study. A total of 420 patients, 34 men (8%) and 386 women (92%), with a mean age of 55 (±14) years, and 606 nodules which had FNA cytological examination, were included in our study (Figure 1). Of the patients, 64% were overweight or obese, with a mean BMI of 28 ± 6. All patients were clinically and biologically euthyroidic, without antithyroid autoantibodies. Of the patients, 72% had a history of goiter or nodule and 13% had undergone thyroid surgery. Most of them had no history of dysthyroidism or cervical irradiation, and were without a family history of thyroid pathology. In the study, 80% of patients were referred by their general practitioner (GP), 14% had undergone a scintigraphy and 6% had cervical CT performed in town.

### 3.2. Description of the Population with Thyroid Cancer

Of the patients, 192 (46%) underwent surgery, which allowed to determine the histological type of 269 nodules. Twenty patients had a malignant histology, but we excluded three patients with extra-thyroid cancers (two metastases from lung cancer and one parathyroid cancer). Finally, we included 17 patients (9%) with thyroid cancer, including 1 bifocal cancer (a total of 18 malignant nodules). The patients of thyroid cancer were exclusively women, with an average age of 50 ± 15 years and a higher BMI than those with benign histology (*p* < 0.05). The main results are detailed in Table 1.

### 3.3. Description of Thyroid Nodules According to Their Ultrasound, Cytological and Histological Characteristics

Of the 266 operated nodules, 18 (7%) were malignant: 61% papillary microcarcinomas (5 classical papillary types and 6 with follicular architecture) and 39% papillary carcinomas (2 classic papillary types and 5 with follicular architecture).

#### 3.3.1. Ultrasound Features

We analyzed the sonographic criteria found in benign and malignant nodules confirmed by histology (Table 2). An important proportion of nodules with malignant histology were larger than 3 cm, solid, hypoechoic, heterogeneous and with central vascularization. The other ultrasound criteria were not statistically significant for the diagnosis of malignancy (Table 2). Before 2016, the TIRADS classification was not yet used in our department. Thus, the shape and rigidity of nodules were not described on ultrasound reports. Therefore, we were unable to establish correspondence with the currently used TIRADS or EU-TIRADS classifications. However, by cross-referencing the ultrasound characteristics and the cytological results of the nodules with the final diagnosis of thyroid cancer, we found that 67% of the malignant nodules were hypoechoic, and 61% had a vascularization of type III or IV (Table 2).

#### 3.3.2. Cytological Results

The cytological characteristics are described in Table 3 and Table 4. Of 266 operated nodules, 12% were classified as Bethesda I (unsatisfactory cytology), 45% as Bethesda II (benign cytology), 30% as Bethesda III (atypia of undetermined significance), 3% as Bethesda IV (suspected of follicular neoplasia including Hürthe cells neoplasia) and 10% as Bethesda V (suspected of malignancy). No nodules were classified as Bethesda VI (malignant). Thyroid cytology alone had a sensitivity of 22.2% and a specificity of 91.1% for the diagnosis of malignancy, with a positive predictive value of 15.4% and a negative predictive value of 94.2%. The cytological assessment by categories, low and medium risk or high risk for malignancy, is described in Table 5.

## 4. Discussion

The incidence of thyroid cancers is increasing worldwide. In 2020, the global incidence rate per 100,000 persons/years was estimated at 10.1 in women and 3.1 in men, compared with 6.1 and 1.9, respectively, in 2012 [13,14]. In Martinique, the age-standardized incidence rate (according to the world population age per 100,000 persons/year) was 8.5 in women and 1.6 in men from 2012 to 2016, while it was respectively 6.5 and 1.5 in 2002–2006 [15]. However, the incidence of thyroid cancer in Martinique is lower than in mainland France, but the standardized mortality rates (per 100,000 persons/year) were similar to those in mainland France [15]. One of the explanations for this increase in incidence is the improvement of diagnostic methods which allow earlier detection of thyroid lesions. This leads to an increase in the number of suspected nodules, and in parallel to an increase in the number of thyroid carcinomas, particularly microcarcinomas [16]. The study of Joseph-Auguste et al., carried out in Martinique from 2007 to 2010, also showed that women are mostly concerned by thyroid pathology, especially thyroid nodule, in which the prevalence was identical to ours [2]. The patients with thyroid cancer were exclusively women, with an average age of 50 ± 15 years. Generally, women are more affected than men by thyroid cancer, but men have more aggressive forms of cancer with a poorer prognosis [17]. Other studies suggest that one of the indirect risk factors of thyroid cancer is iodine deficiency [18,19]. Iodine is involved in the synthesis of thyroid hormones, and its deficiency would lead to an increase in the volume of the thyroid (goiter) and in the production of TSH. There are no data in the literature on iodine status in the French West Indies. It is very likely that small Caribbean islands surrounded by the ocean are less at risk of iodine deficiency. We therefore assume that the adequate iodine supply in our region could not explain our results. TSH levels were on average normal in both benign and malignant histology groups, contrary to a previous study [20]. However, we observed a high prevalence of goiter in our study, estimated in 265/420 (63%) of cases. In discordance with the previous study of Rago et al. [21], but in agreement with the study of Schiffmann et al. [20], patients with malignant histology in our study were more frequently carriers of a multinodular goiter (71%) than a single nodule (*p* > 0.05). However, one of the main etiologies of goiter is iodine deficiency [18]. In view of these results, we hypothesize that our findings cannot be explained by iodine status and that other factors, including environmental and genetic factors, may be suspected [6,22].

In our study, the patients with thyroid cancer were overweight or obese, with a mean BMI of 30 ± 7. A high BMI was found more frequently in patients with malignant histology (*p* < 0.05). The KANNARI survey conducted in Martinique from 2013 to 2014 and the PODIUM study showed a significant prevalence of obesity and overweight in the West Indies territories. These cohorts have shown that almost 60% of people over the age of 16 are overweight, and that 28% of individuals in these regions are affected by obesity [23,24]. The prevalence of obesity is increasing in Martinique, especially among women (32.9%). Previous studies [19,25,26,27,28] have shown a statistically significant link between obesity and thyroid cancer, particularly in women [19,25,27].

The majority of cancer patients were from the North Atlantic (53%), 35% from the Central Atlantic and 12% from the South Atlantic. No patient was from the North Caribbean. We did not find any significant difference in the location of patients with benign or malignant histology (*p* > 0.05). The geographical distribution of cancers seems to be similar to that found in a previous study [2]. According to the Martinique Directorate of Food, Agriculture and Forestry, the island of Martinique, and particularly the North Atlantic region, is affected by chlordecone soil pollution [29]. Chlordecone is a persistent organic pollutant (POP), and an endocrine disruptor, which could interfere with estrogenic effects [30,31,32]. A prospective study with blood measurements of chlordecone in cancer patients could allow studying this hypothesis as well as the relationship between this pesticide and thyroid cancer.

In our study, none of the standard ultrasound criteria for malignancy were statistically significant for thyroid cancer. This may be due to the small cancer sample in our study. Hypoechogenicity was frequently found in malignant nodules, in agreement with the literature [2,33,34]. However, we did not find the irregular contour parameter as a predictive factor for thyroid cancer [33].

The absence of significant ultrasound criteria for thyroid cancers in our study could be explained by the large number of papillary microcarcinomas, in 11/18 cases. The microcarcinomas are indeed difficult to detect by ultrasound.

In our study, the predominant histological type was papillary carcinoma, in agreement with the literature [2,35,36]. However, this is contrary to the study of Finlayson et al. [37], in which the incidence of follicular carcinoma was higher in Afro-Caribbean persons living in England. It should be noted that follicular carcinoma is more frequently found in areas with iodine deficiency [18].

Moreover, 61% of the cancers were papillary carcinomas of vesicular architecture (including microcarcinomas). Our results are similar to previous studies [38,39] that noted the absence of irregular contours, halo and microcalcifications in the nodules, which turned out to be papillary carcinomas of vesicular architecture, unlike those of the classical histological type. These nodules appeared to have benign presentations. At the time of our study, non-invasive follicular neoplasm with nuclear features (NIFT-P) was not described. In 2016, papillary carcinoma of encapsulated vesicular architecture was reclassified as NIFT-P due to its indolent nature, good prognosis and low risk of recurrence [40]. Taking into account this new entity in the re-evaluation of the Bethesda classification 2017, the malignancy rates have thus decreased for categories III, IV and V [5]. NIFT-P is usually treated by lobectomy and not by thyroidectomy without radioactive iodine, which decreases the risk of complications related to these treatments [40]. Ultrasound features in this setting appear to be insufficient to assess the risk of malignancy in our region.

### Bethesda System 2010 and Prediction of Malignancy: Are the Standard Criteria Applicable to the French Afro-Caribbean Population?

At the time our patients were explored, the Bethesda system 2010 [4] was the gold standard for classifying nodules according to risk of malignancy and deciding on management [6,41]. However, a benign cytology does not formally exclude malignancy. Only histological analysis provides a definitive diagnosis.

Of 266 operated nodules, 12% had unsatisfactory cytology (Bethesda I), 45% benign cytology (Bethesda II), 30% atypia of undetermined significance (Bethesda III), 3% suspected of follicular or Hürthe cells neoplasia (Bethesda IV) and 10% suspected of malignancy (Bethesda V). No nodules were classified as Bethesda VI (malignant).

The cytological results were not confirmed by histology in 15% of cases, results close to the 11% of the previous study of Joseph-Auguste et al. [2]. The predictive value of the Bethesda system in the risk of malignancy in our population is therefore questionable.

Ideally, the Bethesda I cytology rate should not exceed 10% [4,5]. In our study, 17% of overall FNAs were classified as Bethesda I: 12% of operated nodules were classified in this category, 9% of which proved to be malignant after histological analysis. A meta-analysis found more malignant nodules by histological examination that were previously classified as Bethesda I (16.8%), but the percentage of operated nodules to obtain this result was less important (8.3%) [42]. This high rate of Bethesda I results on FNA may be due to the sampling conditions [4]. Woo et al. [43] showed that hypoechogenicity was more frequently found in unsatisfactory cytology. A decrease in ultrasound penetration due to high cell density could result in difficult sampling conditions and inconclusive results. In addition, Chung et al. [44] suggest that a large nodule with a thick capsule would prevent optimal sampling due to poor needle penetration. In view of the fact that hypoechogenicity and a size greater than 3 cm are features of the majority of benign and malignant nodules, we question their explanatory role in our inconclusive results. For this reason, for nodules initially classified as Bethesda I, it is recommended to recheck and to repeat the cytological examination in order to obtain better results, especially in the case of solid nodules [4,5,6].

In our study, 2.5% (3/119) of the operated nodules classified as Bethesda II turned out to be malignant by histological analysis. According to the literature [4,5], the false-negative rate in Bethesda II cytology is low, estimated to be between 0 and 3%. Therefore, our results are in agreement with the literature. We found fewer discordant results in Bethesda II than the meta-analysis of Bongiovanni et al., who reported a rate of 3.7% [42]. In this category, clinical and ultrasound surveillance is usually proposed [4,6]. However, in our study, 119/333 (36%) nodules classified as Bethesda II were operated on. We can therefore affirm that the criteria for operability were not only based on cytological results but that other factors were taken into account (size of the nodule or goiter, functional discomfort, clinical signs, etc.).

In general, the proportion of thyroid cytology classified as Bethesda III should not exceed 7–10% [4,5]. In our study, it was 21%, a result similar to the previous studies of Archuletta et al. and Yoon et al., but higher than that of Joseph-Auguste et al., a study that reported a rate of 9.5% [2,34,45]. Pre-analytical factors such as the conditions of sampling, fixation and conservation of cytological material could explain the preponderance of this category [4]. Since the Bethesda III classification includes a variety of difficult-to-interpret lesions that cannot be categorically classified as benign or malignant, or obviously suspicious of malignancy, this may also be responsible for the over-diagnosis of this category [4,5,46]. We had 9% of operated nodules that turned out to be malignant on histology, a result in agreement with previous studies [5,45].

In our study, Bethesda III was the most represented category of malignancy, because 39% (7/18) of thyroid cancers had nodules initially classified as Bethesda III. Therefore, special attention should be paid to these Bethesda III nodules in our population. Usually, a follow-up at six months is recommended in this category [4]. Complementary molecular biology or repeat cytology may also be useful in some cases.

In our study, few nodules were classified as Bethesda IV (1%): 12.5% (1/8) of the operated nodules in this category had malignant histology, which is half the rate of the meta-analysis by Bongiovanni, who found a rate of 26.1% [42]. It is estimated that about 15–30% of thyroid cytology classified as Bethesda IV are usually malignant [4,5]. Thus, our observed 12.5% rate is lower than expected [2]. In general, when the cytology result is classified as Bethesda IV, follow-up is recommended in order to rule out a possible follicular neoplasm. In case of identical results on two consecutive occasions, lobectomy with frozen section examination is recommended [4,5,6].

In the Bethesda V category, 26/33 (79%) nodules were operated on. According to the recommendations [6], all of them should be surgically checked. Among the non-operated Bethesda V nodules, two were of extra-thyroidal origin (excluded from the analysis) and the others belonged to patients who were not operated on in Martinique and for whom we did not find an operative or histological analysis report.

Only 15% of operated nodules with Bethesda V have a malignant histology. These rates remain low and raise questions about the value of the Bethesda system as a predictive factor of malignancy.

The cytological diagnosis made by general pathologists not specialized in thyroid cytology could explain the absence of the category VI of the Bethesda system for thyroid cytology (malignant). This may also explain the high number of cytology classified as Bethesda III (atypia of undetermined significance).

In our study, the evaluation of cytology based on the Bethesda system found a sensitivity of 22.2%, a specificity of 91.13%, a positive predictive value of 15.3% and a negative predictive value of 94.1% (Table 5), which represents a significant difference compared to the previous study in Martinique, which found 83%, 72%, 3% and 97%, respectively [2]. The sensitivity of thyroid cytology in our study was also lower than previous studies [42,47].

It is important to remember that in our study, the Bethesda I, II, III and IV were considered as statistically low and medium risk for the diagnosis of malignancy, and only the V and VI categories were considered as high risk. Therefore, the number of high risk of malignancy was low compared to the total number of cancers (4/18), which leads to a low sensitivity. In addition, the rate of false negatives was high compared to the number of cancers (14/18).

In view of these results, the Bethesda system does not seem to be a reliable indicator of the probability of malignant nodules in our study. However, according to the literature, we found a high specificity of Bethesda system cytology, at 91.13% [2,42,47]. We can therefore conclude that the Bethesda system was a good tool for determining nodules with benign histology in our population.

In our study, ultrasound alone or cytology alone cannot predict malignancy. By correlating the ultrasound and cytological data of the malignant nodules (Table 3), we observed that they were mostly hypoechoic with type III or IV vascularity and that their cytology results were mainly classified as Bethesda III and V. Thus, in our population, particular attention should be paid to vascularized hypoechoic nodules with a cytology classified as Bethesda III and V, for which the number of cancers was high compared to the literature.

The 2010 Bethesda system was revised in 2017 [4,5]. Changes were made to better discriminate malignant from benign lesions. In addition to the removal of NIFT-P from the category of papillary carcinomas due to its indolent nature and good prognosis, the 2017 Bethesda system also introduced the use of molecular tests in case of Bethesda III or IV cytological results in order to decrease heavy surgical management.

## 5. Conclusions

Here, we specified epidemiological and clinical criteria predictive of thyroid malignancy, which could sensitize and guide general practitioners in their diagnostic approach in the context of a mostly Afro-Caribbean population. In this study, most nodules were benign, often requiring simple surveillance with attention to any subsequent clinical change. The prevalence of goiters in our study was also high. However, we found that thyroid cancers were most often observed in obese women in their 50s.

In our experience, hypoechogenicity associated with central vascularity were the most common sonographic findings in malignant nodules. In this study, thyroid cytology graded Bethesda IV or V was less accurate than expected for the prediction of malignancy. However, the other categories, especially Bethesda I, should not be neglected, because its risk of malignancy in our cohort was as high as Bethesda III. For this reason, we recommend a second cytology for nodules initially classified as Bethesda I and a confrontation with clinical and ultrasound data for nodules classified as Bethesda IV and V in order to eliminate any risk of malignancy and adapt patient management. In our mostly Afro-Caribbean population, papillary microcarcinoma was the most frequent histological type, which may explain the inconclusive ultrasound and cytological results. Further prospective studies could refine the clinical and sonographic criteria predictive of malignancy in our population. Special attention should be paid to soil pollution by organochlorine pesticides in Martinique, including chlordecone. The relationship between chlordecone and cancer is currently being explored.

## Figures and Tables

**Table 1:** Characteristics of patients by histological findings.

Variables	Benign Histology (%) *n* = 172	Malignant Histology (%) *n* = 17	*p*
**Age** (years)	53 ± 12	50 ± 15	0.19
**Sex**			0.22
Femal	152 (88)	17 (100)	
Male	20 (12)	0 (0)	
**Geographical location**			0.52
North Atlantic	86 (50)	9 (53)	
North Caribbean	4 (2)	0 (0)	
South	43 (25)	2 (12)	
Center	39 (23)	6 (35)	
**Personal history of thyroid disease**			
No	47 (27)	3 (18)	0.57
Surgery	20 (12)	1 (6)	0.70
Hypothyroidism	13 (8)	0 (0)	0.61
Hyperthyroidism	9 (5)	3 (18)	0.08
Nodule/goiter	119 (69)	14 (82)	0.40
**History of cervical irradiation**			NA
Yes	0 (0)	0 (0)	
No	172 (100)	17 (100)	
**Family history of thyroid disease**			0.20
Yes	38 (22)	1 (6)	
No	134 (78)	16 (94)	
**BMI kg/m^2^**	27 ± 6	30 ± 7	0.04
**BMI kg/m^2^**			0.29
Less than 18	6 (3)	0 (0)	
Between 18 and 25	61 (35)	3 (18)	
More than 25	105 (61)	14 (82)	
**Clinical dysthyroidism**			0.20
Yes	35 (20)	1 (6)	
No	137 (80)	16 (94)	
**Number of nodules on clinical examination**			0.45
Unique	69 (40)	5 (29)	
Multiple	103(60)	12 (71)	
Absence	0 (0)	0 (0)	
**Adenopathies**			0.79
Presence	53 (31)	6 (35)	
Absence	119 (69)	11 (65)	
**TSH miU/L** (Standard 0.27–4.20 miU/L)	1.05 ± 0.79	1.06 ± 0.87	0.52
**FT4 pmol/L** (Standard 12–22 pmol/L)	12.15 ± 3.20	10.76 ± 2.35	0.15
**Anti-thyroid autoantibodies**			0.01
Negatives	156 (91)	11 (64)	
Anti-TGB and/or TPO-positive	12 (7)	4 (24)	
TRAK-positive	1 (1)	1 (6)	

**Table 2:** Comparison between the main characteristics of thyroid nodules and the final histological diagnosis (benign and malignant).

Variables	Benign Histology *n* = 248 (%)	Malignant Histology *n* = 18 (%)	*p*
**Echogenicity**			0.09
Hypoechogenic	167(67)	12 (67)	
Isoechogenic	76 (40)	4 (22)	
Hyperechoic	5 (2)	2 (11)	
**Contour**			1
Regular	214 (86)	16 (89)	
Irregular	34 (14)	2 (11)	
**Microcalcifications**			1
Presence	24 (10)	1 (6)	
Absence	224 (90)	17 (94)	
**Location**			0.16
Isthmus	17 (7)	2 (11)	
Right lobe	112 (45)	11 (61)	
Left lobe	119 (48)	5 (28)	
**Content**			1
Liquid	2 (1)	0 (0)	
Solid	192 (77)	14 (78)	
Mixed	54 (22)	4 (22)	
**Size**			0.69
Less than 1 cm	5 (2)	0 (0)	
Between 1 and 2 cm	50 (20)	5 (28)	
Between 2 and 3 cm	72 (29)	6 (33)	
More than 3 cm	121 (49)	7 (39)	
**Structure**			0.44
Homogenous	81 (33)	4 (22)	
Heterogeneous	167 (67)	14 (78)	
**Vascularization**			0.41
Non-vascularized	21 (9)	0 (0)	
Type 1	16 (6)	1 (5)	
Type 2	60 (24)	5 (28)	
Type 3	131 (53)	10 (56)	
Type 4	18 (7)	1 (6)	
Vascularized without precision	2 (1)	1 (5)	
**Halo**			0.35
Presence	5 (2)	1 (6)	
Absence	243 (98)	17 (94)	

**Table 3:** Correspondence between Bethesda system 2010 and ultrasound characteristics in the cancer group. Number (N) and percentage (%) of nodules in each category being hypoechoic or having irregular contours, type III or IV vascularization, halo and microcalcifications.

Bethesda 2010	HypoechogenicN (%)	Irregular Contours	Type III or IV Vascularization	Halo	Microcalcification	Total Cancer
I = Not diagnosed or unsatisfactory	0 (0%)	1 (33%)	3 (100%)	1 (33%)	0 (0%)	3
II = Benign	3 (100%)	0 (0%)	1 (33%)	0 (0%)	0 (0%)	3
III = Atypia of undetermined significance	5 (71%)	1 (14%)	3 (43%)	0 (0%)	0 (0%)	7
IV = Suspected follicular neoplasia (including Hürthle cell neoplasia)	1 (100%)	0 (0%)	1 (100%)	0 (0%)	1 (100%)	1
V = Suspected malignancy	3 (75%)	0 (0%)	3 (75%)	0 (%)	0 (%)	4
VI = Malignant	0	0	0	0	0	0
Total	12 (67%)	2 (11%)	11 (61%)	1 (6%)	1 (6%)	18

**Table 4:** Bethesda system 2010—Thyroid cytology and final histology results of thyroid nodules. Number (N) and percentage (%) of thyroids nodules which have Bethesda system FNA cytology compared to operated cases and final histological analysis in each category.

		Thyroid FNA Cytology N (%)	Nodules Operated N (%)	Benign Histology N (%)	Malignant Histology N (%)
Bethesda system 2010, Thyroid cytology	Not diagnosed or unsatisfactory (I)	104 (17)	33/104 (32)	30/33 (91)	3/33 (9)
	Benign (II)	333 (55)	119 /333(36)	116/119 (97.5)	3/119 (2.5)
	Atypia of undetermined significance (III)	127 (21)	80/127 (63)	73/80 (91)	7/80 (9)
	Suspected follicular neoplasia (including Hürthle cell neoplasia) (IV)	9 (1.5)	8/9 (89)	7/8 (87.5)	1/8 (12.5)
	Suspected malignancy (V)	33 (5.5)	26/33 (79)	22/26 (85)	4/26 (15)
	Malignant (VI)	0	0	0	0
	Number of nodules	606	266	248	18

**Table 5:** Cytology assessment by categories of risk for malignancy (low and medium risk, and high risk of malignancy).

	Malignant Histology	Benign Histology	Total
High risk of malignancy cytology (Bethesda V)	4	22	26
Low or medium risk of malignancy cytology (Bethesda I, II, III, IV)	14	226	240
Total	18	248	266

## Data Availability

Data are available in the manuscript. Additional data can be obtained upon reasonable request to the corresponding author.
